# On the NS-DSSB unidirectional estimates in the SAMPL6 SAMPLing challenge

**DOI:** 10.1007/s10822-021-00419-0

**Published:** 2021-10-09

**Authors:** Piero Procacci, Marina Macchiagodena

**Affiliations:** Department of Chemistry, University of Florence, Via Lastruccia n. 3, 50019 Sesto Fiorentino, FI Italy

**Keywords:** SAMPL6, Binding free energy, Non-equilibrium, Crooks theorem, Jarzynski identity, Double-system/single-box

## Abstract

In the context of the recent SAMPL6 SAMPLing challenge (Rizzi et al. 2020 in J Comput Aided Mol Des 34:601–633) aimed at assessing convergence properties and reproducibility of molecular dynamics binding free energy methodologies, we propose a simple explanation of the severe errors observed in the nonequilibrium switch double-system-single-box (NS-DSSB) approach when using unidirectional estimates. At the same time, we suggest a straightforward and minimal modification of the NS-DSSB protocol for obtaining reliable unidirectional estimates for the process where the ligand is decoupled in the bound state and recoupled in the bulk.

**Supplementary Information:**

The online version contains supplementary material available at 10.1007/s10822-021-00419-0.

## Introduction

In Ref. [1], in the context of the SAMPL6 challenge [2], the reliability and efficiency of absolute binding free energy (ABFE) calculations in host-guest systems was systematically assessed using various molecular dynamics (MD) techniques, including the reaction coordinate-based attach-pull-release scheme (APR) [3], the alchemical double decoupling approach (DDM) [4] with or without $\lambda$-hopping [5] and a nonequilibrium alchemical switching method termed “double system single box” (NS-DSSB) [6]. Surprisingly, the results suggested that specifying force field parameters and partial charges was insufficient to generally ensure reproducibility. Differences in the computed ABFE up to $\simeq 4$ kcal/mol were observed between seemingly converged predictions, even with almost identical simulations parameters and system setup (e.g., Lennard-Jones cutoff, ionic composition). The differences between the methods were higher when analyzing the CB8-quinine system where all methodologies significantly overestimated the binding affinity, with APR yielding the best result of − 10.5 kcal/mol compared to the experimental counterpart of − 6.5 kcal/mol [7]. In the CB8-quinine system, the nonequilibrium switching (GROMACS/NS-DSSB) obtained the overall highest efficiency with an ABFE prediction of − 11.3 kcal/mol, close to the APR result but overestimating the binding strength by nearly 5 kcal/mol corresponding to more than three order of magnitude in the dissociation constant.

The NS-DSSB ABFE was computed by way a nonequilibrium (NE) *bidirectional* approach using two series of nonequilibrium simulations. In the *forward* process the bound ligand was decoupled while an unbound ligand in the bulk was recoupled in the same MD box, and in the *reverse* process the *restrained* bound guest was recoupled and the distal bulk guest decoupled. These two NE processes were performed using a time inverted protocol recovering the ABFE as the crossing point of the (symmetric) forward $P_f(-W)$ and reverse $P_r(W)$ work distributions via the Crooks theorem (CT) [8] implemented using the Bennett Acceptance Ratio (BAR) [9, 10]. Despite the need of a double full calculation, in case of the CB8-G3 system, the bidirectional NS-DSSB was found to converge to a stable and precise ABFE value investing a total simulation time that was on the average less than half of that of other equilibrium-based techniques. Unidirectional estimates in NS-DSSB, based on the so-called “Gaussian approximation” or using the Jarzynski exponential average, were also tested yielding estimates heavily dependent on the duration of the nonequilibrium switches and, in some cases, in strong disagreement with the BAR estimate.

In this contribution we will show that the reason for the severe errors of the unidirectional estimates in the NS-DSSB nonequilibrium approach tested in the SAMPL6 SAMPLing challenge [1] lies in the large dissipation of the process that *includes* the restraint potential. In fact, besides the alchemical work, the NE processes in NS-DSSB involves also the switching off (or on) of the restraint intermolecular potential used in the decoupled (or coupled) bound state [11]. This “restraint work” adds up to the total NE work as a huge contribution, significantly widening the work distribution thereby preventing the determination of reliable unidirectional estimates. We also show that better and more stable unidirectional estimates can be obtained for the case of the *forward* NS-DSSB process by simply lifting the restraint potential and accounting for the standard volume correction by evaluating the binding site volume in the unrestrained fully coupled bound state.

## Methods

Here we focus on the challenging CB8-quinine host-guest system. The CB8 host (Cucurbit[8]uril) is a 144 atoms toroidal macrocyclic molecule made of glycoluril monomers linked by methylene bridges [7]. The quinine guest, a well known antimalarial drug, is a bulky molecule (49 atoms) characterized by a methoxy quinoline moiety functionalized by an ethenyl-1-azabicyclo[2.2.2]octan-2-yl group. Both CB8 and quinine pose important computational challenges, due to sampling issues related to the low frequency torus deformation modes in the host and to the size and conformational activity of the guest. Structural details of CB8 and quinine can be found in Ref. [1] as well as on the dedicated SAMPL6 GitHub site [2].

**Fig. 1:**
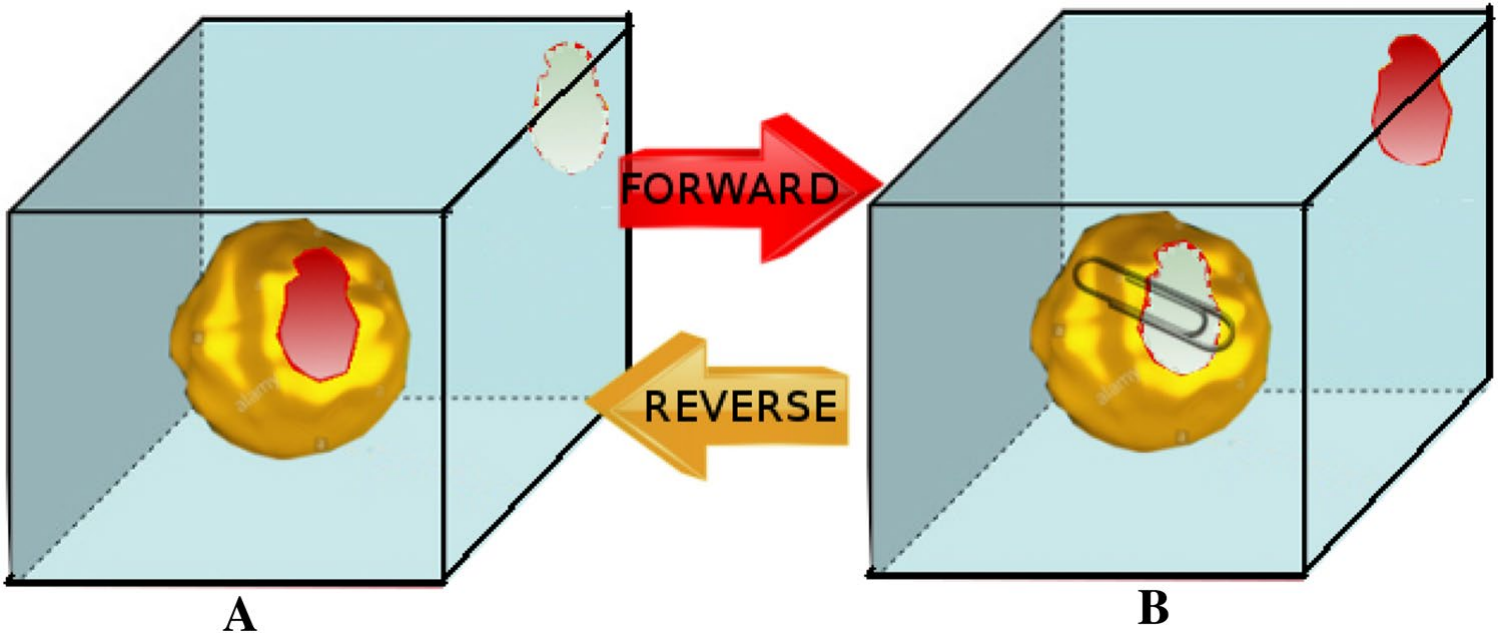
Schematic representation of the NS-DSSB approach. The clip on the right (state B) indicates the presence of a restraint potential

The NS-DSSB method is an alchemical nonequilibrium technique that was first described in Ref. [6]. The method implements, in essence, a nonequilibrium variant of the alchemical thermodynamic cycle based on Free Energy Perturbation (FEP) on the so-called $\lambda$-stratification [12] for ABFE determination. In NS-DSSB, the initial end-states are sampled using replicates of conventional equilibrium MD simulations (for a total time of hundreds of ns) in a MD box with one ligand bound to the host and the other kept in the bulk solvent (represented by TIP3P [13] water molecules). The two thermodynamic end-states are characterized by a coupled and a decoupled ligand. In the state A, the guest in the bulk is decoupled while the (unrestrained) bound guest is fully coupled. In the state B the bound guest is decoupled and kept in the CB8 toroidal cavity by a host-guest restraint potential while the guest in the bulk is fully coupled. The thermodynamic cycle can be completed in the two senses (A to B or B to A), by connecting the end-states by a swarm of NS trajectories where the two ligands are rapidly (few ns at most) and simultaneously decoupled or recoupled and evaluating, on each of these trajectories, the work done by the driven alchemical coordinate *and* the cost of gradually imposing/releasing the restraint potential. The NS-DSSB is schematically illustrated in Fig. 1

In Ref. [1], state A an B were sampled using up to 5000 configurations, producing an equivalent number of NS trajectories in each sense, each lasting up to 2 ns. The technique can provide in principle in both directions a direct estimate of the *dissociation* free energy (A to B) or of the *binding* free energy (B to A) by using the Jarzynski exponential average [14] on the collection of forward or reverse work values. This estimate must be corrected by the standard state dependent term related to the guest-host restraint potential. In Ref. [1], unidirectional estimates (and notably those based on the “Gaussian approximation”) turned out to be unreliable, even when using relatively long NS trajectories. Further technical details on the NS-DSSB methodology can be found in Ref. [1].

## The Boresch-style restraint potential

We shall now analyze more in-depth the restraint potential used in the end-state B of the NS-DSSB SAMPL6 SAMPLing submission. The approach, originally introduced by Karplus and co-workers [11] in the context of alchemical FEP, was proposed to prevent the “wandering [decoupled] ligand” effect and to further limit the relative host-guest orientational motion at low coupling in double decoupling calculations [4]. The host-guest restraint potential involves six coordinates, one distance, two bendings and three dihedral angles defined in terms of the atomic coordinates of three host atoms and three guest atoms and chosen so as to orient the guest in a configuration compatible with presumed binding pose. We refer to Fig. 2 of Ref. [11] for details on the definition of the six host-guest intermolecular coordinates.

The alchemically derived binding free energy should then be corrected by a volume term (called $\varDelta A_r$) due to the imposed host-guest coupling restraint potential. According to Ref. [11], the correction $\varDelta A_r$ can be evaluated *analytically* if the partition function of the complex *with the decoupled and restrained* guest can be factorized into partition functions depending on the host coordinates, the ligand coordinates, and the six restraint coordinates which should represent “the external DOFs [degrees of freedom] of the ligand” (see Eq. 11 of Ref. [11]). The resulting analytic correction to the ABFE depends in essence on the product of the six force constants $K_{\mathrm{rstr}}^i$ divided by the restraint distance. If the $K_{\mathrm{rstr}}^i$ (expressed in kcal/mol/Å$^2$, kcal/mol/rad$^2$ and kcal/mol, for stretching, bending and torsional terms, respectively) are chosen all equal (as it is normally done in the practice of ABFE calculations [15, 16]), then the correction (Eq. 14 of Ref. [11]) can be written as:

1 $$\begin{aligned} \varDelta A_r= RT \left[ \ln \frac{V_0}{\pi (RT)^3} - \ln (d_1^2\sin \theta _2 \sin \theta _3 ) + 3 \ln K_{\mathrm{rstr}} \right] \end{aligned}$$

where $V_0=1661$ Å$^3$ is the standard state volume. Eq. 1 is independent of the three equilibrium dihedral angles while it has a singularity when one of the bending angles $\theta _{2,3}$ is equal to zero.

The Boresch-style restraints should be handled with care as an unattentive choice of the six intermolecular coordinates can lead to systematic errors (see section 1 and Fig. S1 in the ESI). The six restraints shown in Fig. 2 of Ref. [11], in particular, should involve triplets of atoms on the host and on the guest that are part of relatively *rigid* moieties. In the NS-DSSB approach reported in the SAMPL6 SAMPLing challenge, the six atoms involved in the restraint potential were judiciously selected. For the host, three atoms on one of the planar glycoluril moieties were chosen, and for the guest, three atoms on the rigid azabicyclo-octanyl moiety were chosen (see data in the ESI.zip archive for details).

**Fig. 2:**
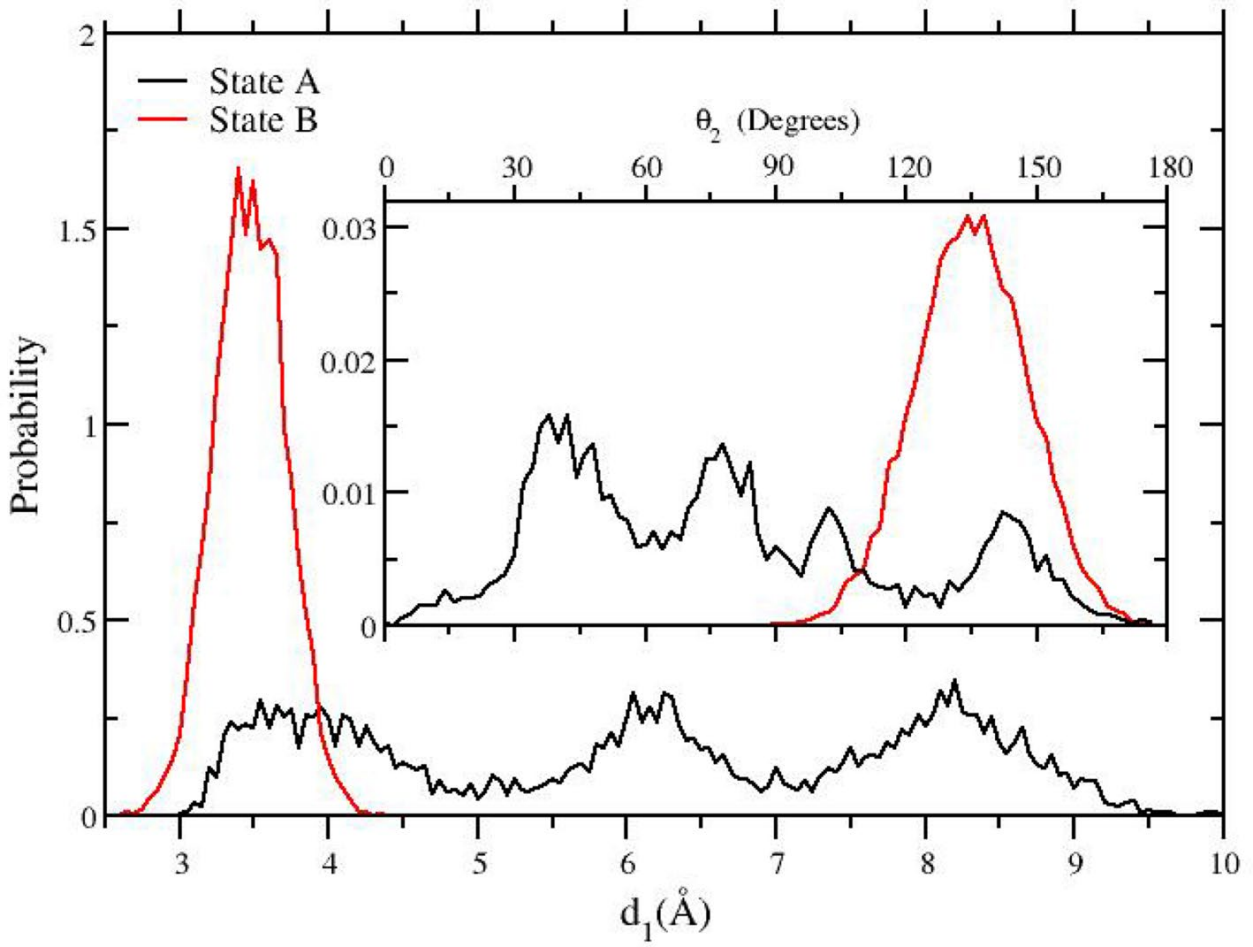
Probability distributions of the $d_1$ distance and $\theta _2$ angle in state A (no restraint and fully coupled bound ligand) and in state B (decoupled ligand with Boresch restraints) where the Boresch-style restraints are enforced on $d_1$ and $\theta _2$ (see ESI for details on the host and guest atoms involved in the definition of the $d_1$ and $\theta _2$ intermolecular coordinates). The histograms where computed using the 5000 configurations sampled in the NS-DSSB experiments in state A and B

In the context of the topology-based decoupling/recoupling NS-DSSB implemented in the GROMACS program [6], the Boresch-style restraints are released or enforced *while* the alchemical process is in course. Strictly speaking, this approach is not equivalent to the procedure described in the reference papers [15, 17] where the cost of imposing the restraint potential is computed in a FEP transition *at the end-state* of the complex with the fully coupled guest and not *during* the alchemical process. This issue is not merely technical as in the SAMPL6 SAMPLing ABFE prediction there is a missing entropic contribution due to the ways the restrained pose could have been selected in state B. As in state A the restraints are not enforced, this term does not cancel out as it does when the cost of imposing/releasing the restraint is evaluated in A and B *before* and *after* the alchemical transition [18]. In Fig. 2, we show the probability distribution of the distance $d_1$ and of the angle $\theta _2$ in state A and state B. In state A (no restraints enforced), the probability distributions of the $d_1$ distances and $\theta _2$ exhibits three and four maxima, respectively, showing that the ligand is free to sample various binding poses.

In state B, the $d_1$ and $\theta _2$ restraints limit the sampling to a single pose, with slightly off-centered maxima with respect to those of one of the unrestrained poses for both the $d_1$ and $\theta _2$ coordinates. In going from A to B, the entropy due to multiple poses in A is lost and a strain (or reorganization) energy is involved in the release or enforcement of the restraint.

## Free energy estimates with Boresch-style restraints

### Bidirectional estimates

**Fig. 3:**
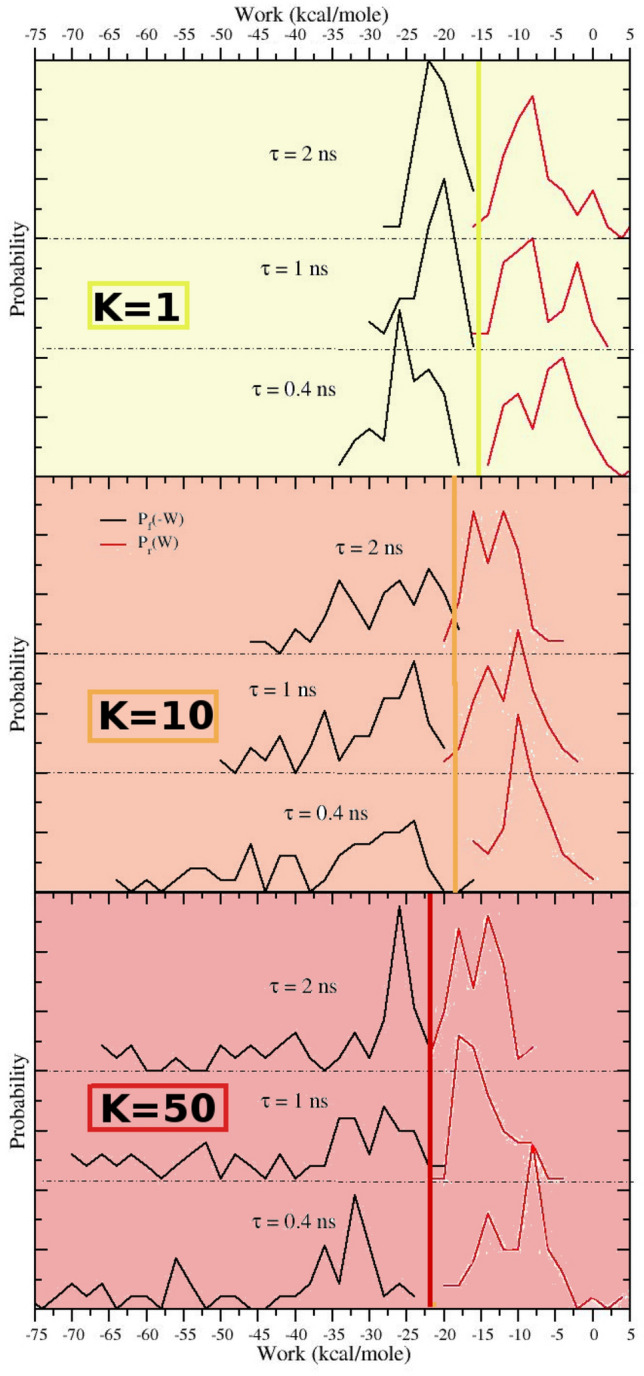
Mirror symmetric forward ($P_F(-W)$, black) and reverse ($P_r(W)$, red) work distributions computed using 50 NS process for various duration times and restraint strengths. In each of the three plots with various restraint strengths, the vertical line corresponds to the BAR estimate of the ABFE obtained with the longest NS time $\tau =2$ ns (see Table 1)

In Fig. 3 we plot the work histograms obtained using 50 forward and reverse NS representative runs (see ESI) of duration of 0.4 ns, 1 ns and 2 ns using a Boresch-style restraint potential with $$K_{\mathrm{rstr}}=1,10,50$$. The work values (in kcal/mol) have been computed from the dhdl.xvg GROMACS-generated files in a single unix command as 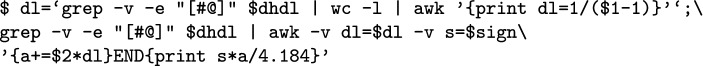 where $ dhdl is the dhdl.xvg filename and $sign=1, $sign=-1 for the forward and reverse process, respectively.

In Table 1, we report the corresponding BAR estimates of the ABFE as the maximum likelihood [10] (ML) crossing point of the two work distributions, corrected using the analytic term $\varDelta A_r$ due to the Boresch-style restraints (see Eq. 1). The 95% confidence intervals have been computed by bootstrapping with resampling over the collection of 50 forward and reverse work values.

**Table 1:** BAR estimates ($\varDelta G_{\mathrm{BAR}}$) and Jarzynski average ($\overline{\varDelta G_{J}} = \varDelta G_J(B\rightarrow A) - \varDelta G_J(A\rightarrow B)$) of the quinine-CB8 standard binding free energy using 50 NS forward and reverse trajectories for various duration times $\tau$ (ns) and restraint strengths (*K*). Free energies are given in units of kcal/mol

$\tau/ns$	$K_{\mathrm{rstr}}$	$\varDelta G_{\mathrm{BAR}}$	$\overline{\varDelta G_{J}}$	$\varDelta A_r$ (Eq. 1)	ABFE
2.0	1.0	$-15.34 \pm 0.69$	$-15.40 \pm 1.49$	3.43	$-11.91 \pm 0.69$
2.0	10.0	$-18.65 \pm 1.01$	$-18.49 \pm 2.14$	7.52	$-11.13 \pm 1.01$
2.0	50.0	$-21.83 \pm 1.09$	$-21.83 \pm 2.39$	10.38	$-11.45 \pm 1.09$
1.0	1.0	$-16.09 \pm 0.98$	$-16.22 \pm 2.26$	3.43	$-12.66 \pm 0.98$
1.0	10.0	$-19.81 \pm 0.61$	$-19.79 \pm 1.39$	7.52	$-12.29 \pm 0.61$
1.0	50.0	$-20.95 \pm 1.97$	$-20.66 \pm 3.97$	10.38	$-10.57 \pm 1.97$
0.4	1.0	$-15.95 \pm 0.73$	$-15.81 \pm 1.50$	3.43	$-12.52 \pm 0.73$
0.4	10.0	$-17.86 \pm 2.43$	$-17.84 \pm 5.39$	7.52	$-10.34 \pm 2.43$
0.4	50.0	$-22.26 \pm 1.46$	$-22.19 \pm 2.75$	10.38	$-11.88 \pm 1.46$

Given the low number of NS trajectories, the ABFE estimates ($\varDelta G_0 = \varDelta G_{\mathrm{BAR}}(K) + \varDelta A_r(K)$) are weakly affected by the duration of the NS experiments and by the strength of the restraints. As shown in Fig. 3, the overlap of the work distributions is limited in all cases and depends weakly on the restraint strength and on the duration NE time for $\tau \ge 1$ ns. Due to such poor overlap, the BAR estimates is in general very close to the arithmetic mean of the forward and reverse Jarzynski average $\overline{\varDelta G_{J}}$ [19].

The estimate of -11.13 kcal/mol, obtained with $\tau =2$ ns and $K_{\mathrm{rstr}}=10$ kcal/mol, is remarkably similar to that reported in Ref. [1] (− 11.3 kcal/mol) with the same setup but computed using 5000 NS runs in both directions. The corresponding 95% confidence interval is 1.01 kcal/mol, about ten times that reported in Ref. [1] using 5000 ns runs, in agreement with the fact that the standard error in the BAR estimate goes as [9, 10] $n^{-1/2}$. The data reported in the Table 1 convincingly demonstrate the robustness of the NS-DSSB *bidirectional* estimate even when using a relatively low number of representative starting A and B points. An investment of 200 ns simulation time is apparently sufficient for recovering the ABFE obtained in the 20 $\mu$s total simulation time used in the 5000 NS trajectories of Ref. [1].

*Precise* (reproducible) estimates, such as the BAR-based bidirectional estimate when using NS-DSSB with Boresch-style restraints, not necessarily imply the same level of *accuracy* [20]. In fact, the selected SAMPL6 SAMPLing simulation protocol introduced an undetected systematic bias. Besides the already cited entropic term due to the restraint imposed only in B (that leads to an overestimate of the dissociation free energy) a second source of ABFE biasing is due to the non perfect overlap of the Boresch-restrained pose with the symmetry-related actual pose (see Fig. 2).

### Unidirectional estimates

While bidirectional estimates (as we have seen) can be precise even for negligible overlap and irrespective of the shape and spread of the work distributions, unidirectional estimates *strongly depends* on the spread and shape of the work distributions. In particular, the accuracy and precision of NE unidirectional estimates are strongly dependent on the *dissipation*, i. e. on the distance between the underlying free energy and the mean NE work values, $\langle W \rangle$. In the SAMPL6 SAMPLing challenges the NS-DSSB participants tested, in each direction, the Jarzynski estimate and the so-called Gaussian approximation:

2 $$\begin{aligned} \varDelta G_{\mathrm{Jar}}= & {} -RT \ln \langle e^{-\beta W} \rangle \end{aligned}$$

3 $$\begin{aligned} \varDelta G_{\mathrm{Gauss}}= & {} \langle W\rangle - \frac{1}{2}\beta \sigma ^2 \end{aligned}$$

using a restraint strength corresponding to $K_{\mathrm{rstr}}=10$ kcal/mol. The Jarzynski estimate is notoriously biased especially for low value of *n* and even if the spread of the work distribution is only moderately larger than $k_BT$ [10]. On the other hand, Eq. 3, provides an *unbiased and exact* estimate of the ABFE only when the work distribution is normal. The Crooks theorem implies in this case that the distribution of the inverted process should be normal too and mirror symmetric with respect to the crossing point, which is patently untrue for $K_{\mathrm{rstr}}\ge 10$ (see Fig. 3).

**Fig. 4:**
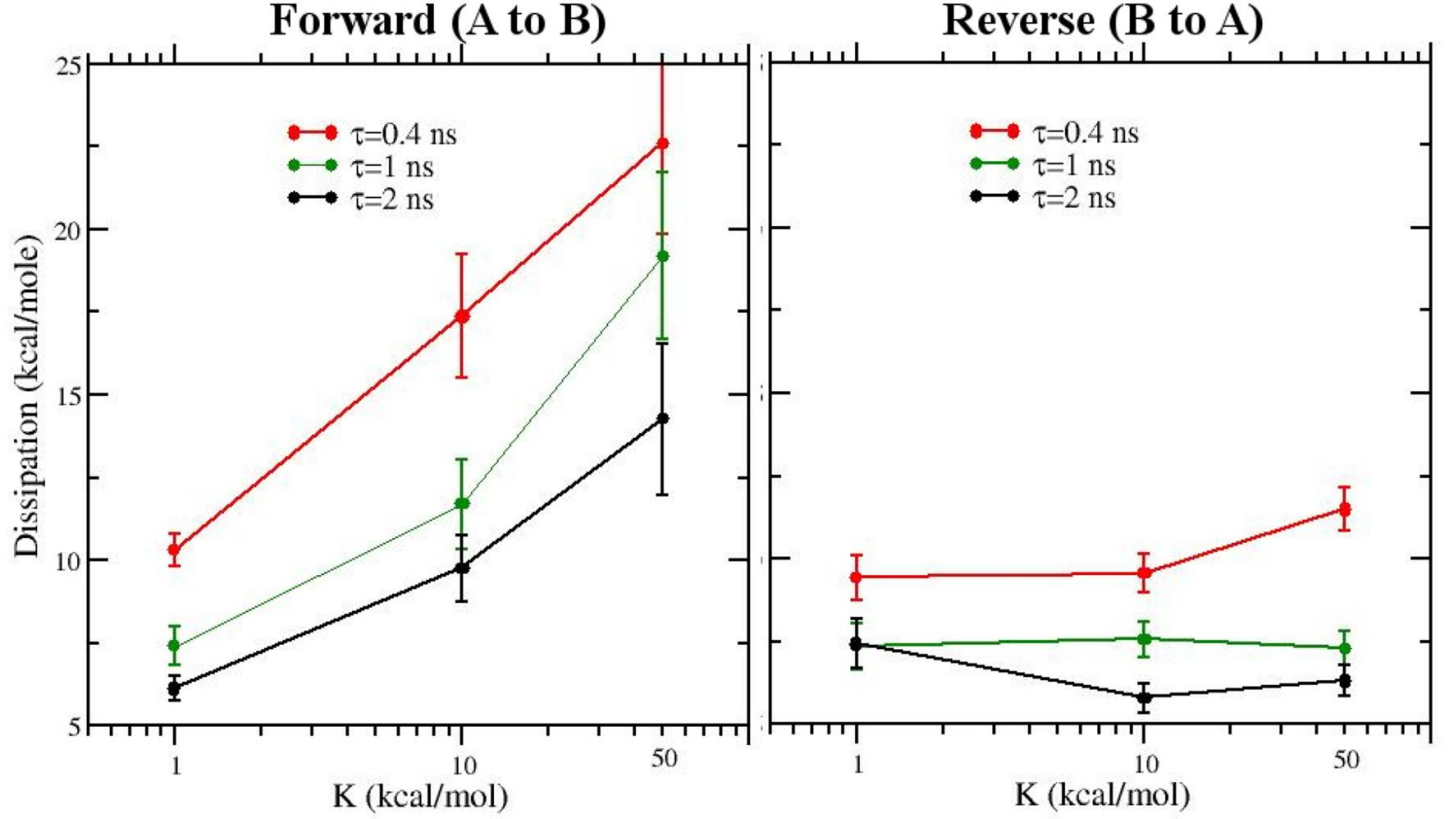
Dissipation in the forward (**a**) and reverse (**b**) direction as function of the restraint strength and of the NE duration time

In Fig. 4 we report the dissipation as a function of the restraint strength and of the duration time of the 50 NS transitions in the forward and reverse NE processes. The forward and reverse dissipation are computed as

4 $$\begin{aligned} W^d_F(K,\tau )= & {} \langle W \rangle _F(K,\tau ) - \varDelta G^0_{BAR}(K) \end{aligned}$$

5 $$\begin{aligned} W^d_R(K,\tau )= & {} \langle W \rangle _R(K,\tau ) + \varDelta G^0_{BAR}(K) \end{aligned}$$

where $\varDelta G^0_{BAR}(K)= 11.3+ \varDelta A_r(K)$ kcal/mol corresponds to the reference value obtained in the SAMPL6 SAMPLing NS-DSSB submission.

From Fig. 4, we note that for the forward (A to B) direction, where the bound ligand is decoupled while being progressively restrained, the dissipation dramatically increases with the restraint strength and with the duration time of the NE transitions, hitting more than 20 kcal/mol for $\tau =0.5$ ns and $K_{\mathrm{rstr}}=50$ kcal/mol. For the reverse direction, conversely, the dissipation shows a moderate increase with the duration time $\tau$ while remaining approximately stable with increasing restraint strength. The asymmetry in the dissipation observed in the forward and reverse direction is due to the inherent asymmetry of the alchemical work *and* of the restraint work. Concerning the latter, the contribution to the dissipation is much less important in the reverse direction (B to A) where the ligand remains bound to one of the symmetrically equivalent poses during the NS transition with an unlikely probability of exploring other equivalent poses in the last stages of the NE process where the restraints are finally released.

When dealing with unidirectional estimates Eqs. 2, 3, we must assume that the work distribution in the opposite direction is unknown. While Eq. 2 can be used in any instance, Eq. 3 can be reliably used only if the distribution is normal. Normality tests are conceived to dismiss (with a certain probability) the null hypothesis (the distribution is normal), but, if passed, they cannot give any certitude on the nature of distribution, especially for poorly resolved histograms as in our cases. For example, all the reverse (B to A) work histograms obtained with $K_{\mathrm{rstr}}=10$ kcal/mol pass the Anderson Darling (AD) normality test [21]. However, as it can be seen from Figs. 3 and 4, the corresponding $K_{\mathrm{rstr}}=10$ kcal/mol reverse distribution $P_r(-W)$ are not symmetric with respect to the ML crossing point and hence, according to the Crooks theorem, these B to A distributions cannot be normal.

**Table 2:** Unidirectional free energy estimates (in kcal/mol) for the restrained CB8-quinine system. All reported free energies are not corrected for the standard state term $\varDelta A_r$ (Eq. 1)

$\tau/ns$	$K_{\mathrm{rstr}}$	Forward		Reverse		BAR
		$\varDelta G_{\mathrm{Gauss}}$	$\varDelta G_{\mathrm{Jar}}$	$\varDelta G_{\mathrm{Gauss}}$ (Eq. 3)	$\varDelta G_{\mathrm{Jar}}$ (Eq. 2)	
2.0	1.0	15.1± 2.2	17.7± 0.6	n/a	− 13.1 ± 1.4	15.34 ± 0.69
2.0	10.0	n/a	20.2 ± 1.8	− 21.2 ± 2.9	− 16.8 ± 1.2	18.65 ± 1.01
2.0	50.0	n/a	24.0 ± 1.3	− 23.3 ± 3.0	− 19.6 ± 2.0	21.83 ± 1.09
1.0	1.0	n/a	18.6 ± 0.8	− 24.0 ± 5.3	− 13.9 ± 2.1	16.09 ± 0.98
1.0	10.0	n/a	22.3 ± 0.9	− 24.4 ± 4.7	− 17.3 ± 1.1	19.81 ± 0.61
1.0	50.0	n/a	22.6 ± 3.7	n/a	− 18.7 ± 1.5	20.95 ± 1.97
0.4	1.0	14.2 ± 4.0	20.4 ± 1.2	n/a	− 11.2 ± 0.9	15.95 ± 0.73
0.4	10.0	n/a	21.2 ± 5.2	− 20.3 ± 4.4	− 14.4 ± 1.3	17.86 ± 2.43
0.4	50.0	n/a	27.1 ± 2.4	− 28.1 ± 6.4	− 17.3 ± 1.4	22.26 ± 1.46

In Table 2 we report the unidirectional estimate Eq. 2 and Eq 3 (when applicable according to the AD test) as calculated from the 50 NS transitions in both directions. The 95% confidence intervals have been estimated by bootstrap with resampling. Expectedly, when compared to the corresponding BAR bidirectional value, the Jarzynski average consistently overestimates the *dissociation* free energy in the *forward* direction and underestimate the *binding* free energy in the *reverse* direction, exhibiting in both cases a *positive* bias. This is so since, for large dissipation, low work trajectories contribute the most the Jarzynski exponential average and low work trajectories are not likely to be sampled effectively using only 50 work values. In the forward direction, because of the huge dissipation (see Fig. 4), the Gaussian estimate is never applicable with the exception of the NS runs with the weakest restraint potential ($K_{\mathrm{rstr}}=1$) for $\tau =0.4$ ns and $\tau =2.0$ ns. In the reverse direction most of the work distributions passes the AD test. The resulting Gaussian estimate Eq. 3, however, is in general imprecise, consistently overestimating the binding free energy with respect to the reference bidirectional value.

What is the source of such huge dissipation observed in the forward direction in the SAMPL6 SAMPLING NS-DSSB setup?

**Fig. 5:**
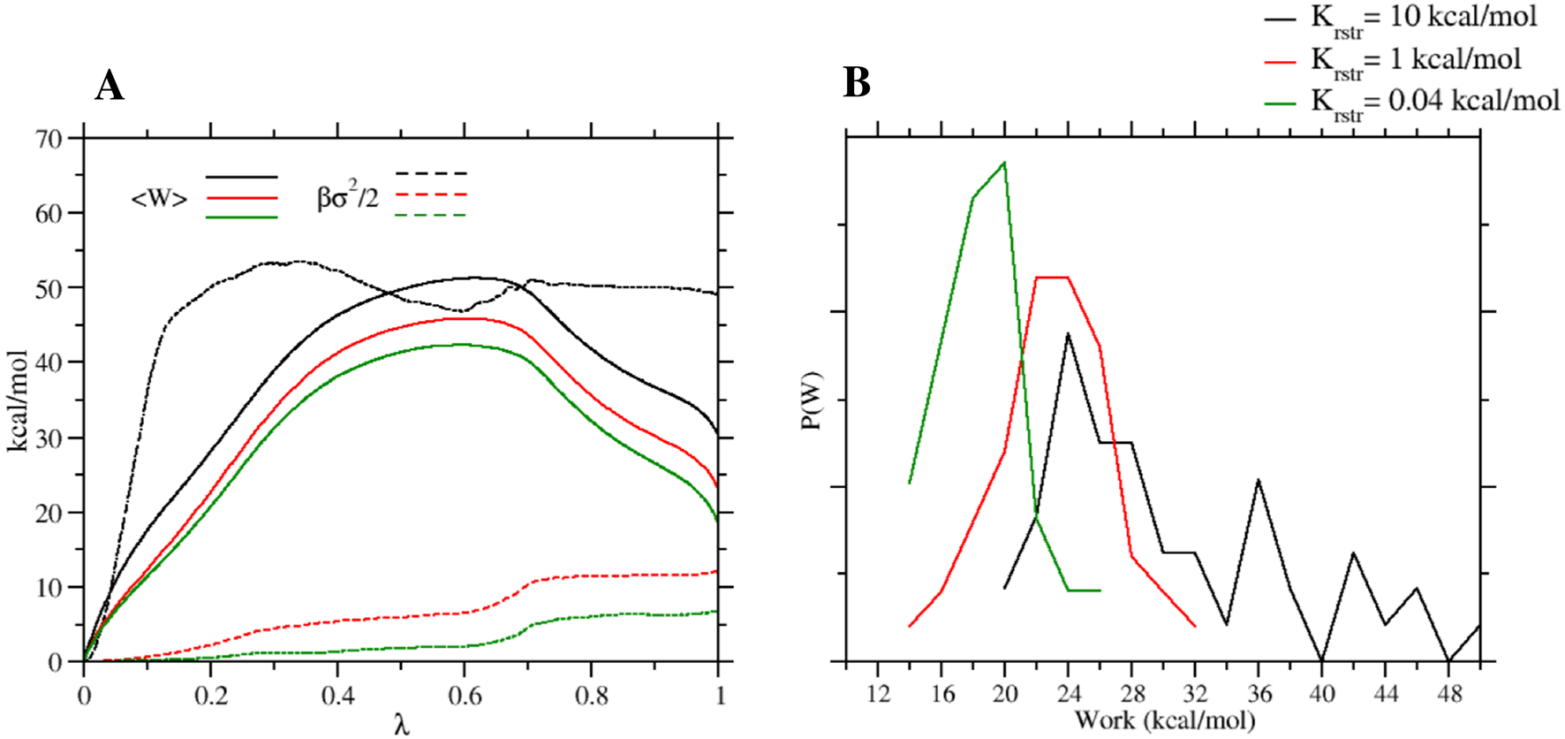
**a** Mean work (solid) and dissipation-related term (dashed) ($\beta \sigma ^2/2$) as a function of $\lambda$ in the forward direction (NS simulations of 1 ns) using various force constant in the Boresch restraint potential. **b** Work probability distribution (50 work values, resolution of 2 kcal/mol) for various force constant in the Boresch restraint potential in the forward direction (NS simulations of 1 ns)

In Fig. 5a, we report the (mean) work along the $\lambda$ coordinate in the forward process for $\tau =1$ ns with the NS-DSSB setup ($K_{\mathrm{rstr}}=10$ kcal/mol), with $K_{\mathrm{rstr}}=1$ kcal/mol and $K_{\mathrm{rstr}}=0.04$ kcal/mol. The mean work (and hence the dissipation) decreases significantly for all $\lambda$ values with decreasing force constant. At the end of the transition, the mean work with $K_{\mathrm{rstr}}=10$ kcal/mol, is more than 10 kcal higher than that obtained with $K_{\mathrm{rstr}}=1$ kcal/mol or $K_{\mathrm{rstr}}=0.04$ kcal/mol. The variance-related energy $\beta \sigma ^2/2$ is also connected to the dissipation (see Eq. 3). We can see that, for the work obtained using the force constant of 10 kcal/mol (used in the original DSSB set-up), the corresponding $\beta \sigma ^2/2$ quantity immediately increases widening the distribution. The NS runs using lower values of the force constant exhibit a much tamer behavior of the variance-related term.

In Fig. 5b, we show the work distributions at $\tau =1$ ns obtained using the three restraint set-up. Here, the effect of the restraint force constant in widening the distribution and in boosting the dissipation is unmistakable. Figures 4 and 5a, b tell us that the extra work due to the enforcement restraint during the forward transition is the main cause of the huge observed dissipation and of the up-shift of the mean work.

Such large dissipation and work up-shift reduce the overlap affecting the BAR estimate and preventing reliable unidirectional estimates in the forward direction especially when using a strong ($K_{\mathrm{rstr}} {\ge } 10$ kcal/mol) restraint as done in the SAMPL6 SAMPLing submission. We note, however, that the forward distributions reported in Fig. 5b get much narrower and Gaussian-like with a weak restraint. What is then the impact of this reduced dissipation for the unidirectional forward estimate of the ABFE when using weak restraints?

## Unidirectional estimates with a weak restraint (forward direction)

When using weak restraints with a large restraint volume $V_\mathrm{rstr}$ (e.g. $K_{\mathrm{rstr}}=0.04$ kcal/mol in Eq. 1 with $V_{\mathrm{rstr}}\simeq 1660$ Å$^3$ or even *no restraint* with $V_{\mathrm{rstr}} = V_{\mathrm{BOX}}$), the unidirectional DSSB estimates in the reverse direction, providing in principle a direct estimate of the *binding* free energy, is constitutively unreliable. In B, we should start the NS transitions from an *equilibrium* high entropy state characterized by a weakly bound *decoupled* ligand where most of the sampled configurations are likely to be quite far from the final stable pose at full recoupling. If we start the NS fast recoupling process from these sub-optimal randomly sampled configurations, we are doomed to end up into fully recoupled NE states that have no resemblance with the real host-guest binding pose, with catastrophic consequences on the predicted ABFE. The estimate in the forward direction does not suffer of these inconveniences. In A, we start in fact with the ligand at full coupling with no restraint, bound to the guest in the most likely pose.

**Fig. 6:**
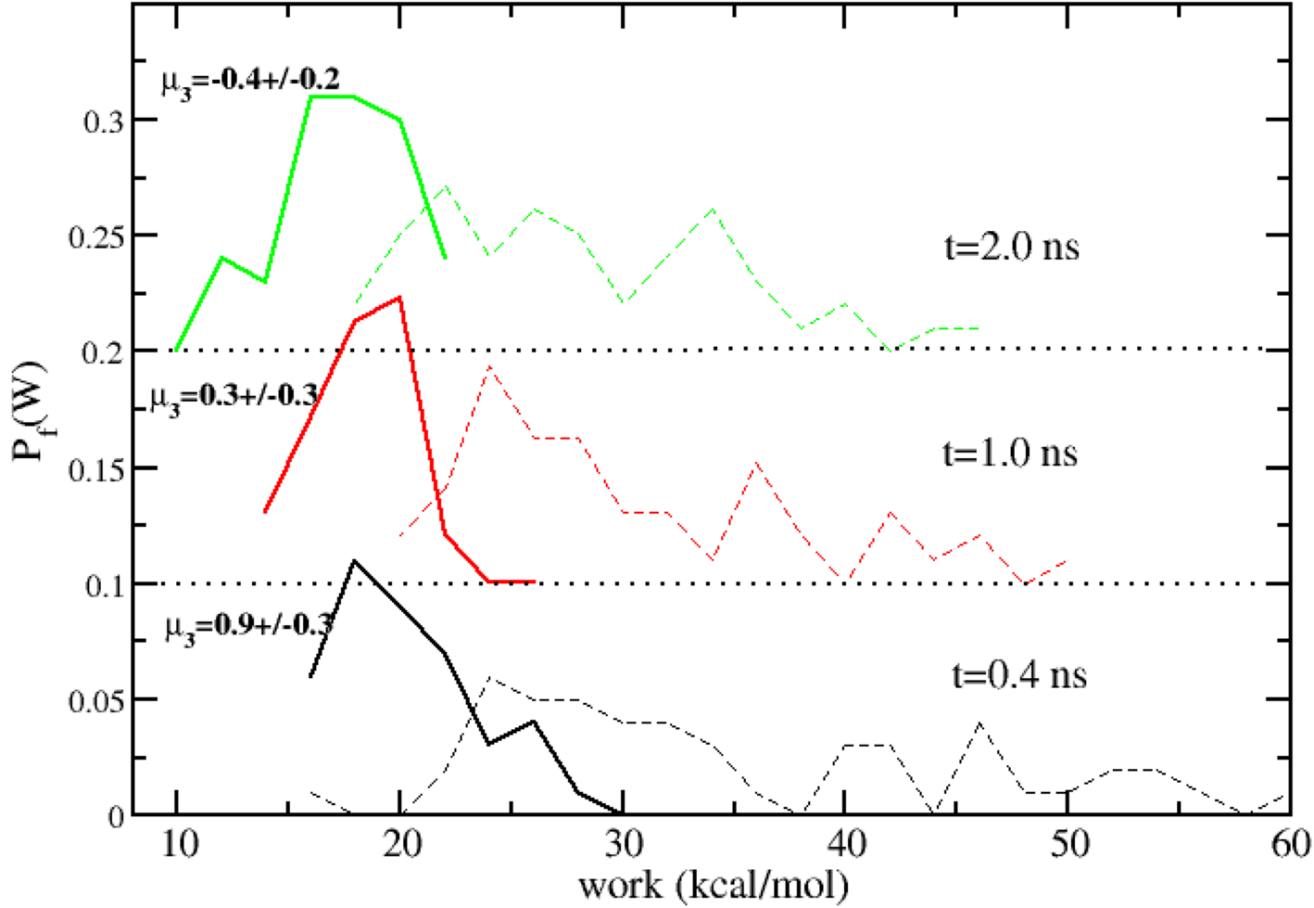
Forward (A to B) work distributions at various NS time with no restraint (solid) and with Boresch-style restraints (dashed). $\mu _3$ is the third standardized moment (or skewness) of the distribution with no restraint

During the equilibrium run in A, the ligand remains in the bound metastable state with an allowance (translational) volume $V_\mathrm{site}$ that can be assessed by examining the host-gust COM-COM distance distribution (see Fig. S2 in the ESI). The ligand ends up in B fully decoupled and with a weak restraint and a corresponding allowance volume, $V_{\mathrm{rstr}}$, that is likely to be larger than the effective binding site volume defining the region of existence of the complex (the so-called indicator function [4]). The standard state correction to the ABFE is given in this case [22, 23] by $\varDelta G_{\mathrm{vol}}= RT \ln (V_0/V_{\mathrm{site}})$. Note that the same correction applies whenever $V_{\mathrm{rstr}} \gg V_{\mathrm{site}}$, hence also when $V_{\mathrm{rstr}}=V_{\mathrm{box}}$, i.e. with the *translational* restraint potential set by the periodic boundary conditions [11]. We recall that the use of strong restraints in ABFE calculations *de facto* implements an estimate of $\varDelta G_{\mathrm{vol}}$, as the difference between the free energy of enforcing the restraint at full coupling and that of releasing the restraint with the decoupled ligand [17, 24].

In Fig. 6 we show work distributions obtained with 50 NS trajectories in the forward directions for various duration of the NE runs with no restraints (solid lines). The dissipation as estimated form the variance is drastically reduced when lifting the Boresch-style restraints (for comparison, we also report with dashed lines the forward distribution obtained using the Boresch-style restraints of the SAMPL6 SAMPLing challenge). Expectedly, the maximum of the work distributions with no restraints moves towards the left (lower dissociation free energy) as the NS time is increased, reflecting the fact that the NE alchemical process is becoming “less irreversible”. On the other hand, the variance of the distribution with no restraint still exhibits a nonlinear behavior with the NE duration time, a clear sign of non normality. All the distributions with no restraints are asymmetric as measured by the reported skewness in Fig. 6, an indication of the existence of multiple poses in A and/or of distinct dissipation routes in the A to B transition. The Jarzynski estimate, on the other hand, is affected (as for the case with restraints (see Table 2)) by a positive bias, amplified by the low number of sampled work values in the right tails of the $P_f(W)$. We can hence try to represents the distributions with no restraints using an alternative unbiased estimate relying on a Gaussian mixture [25]. In the Table 3, we report the unidirectional estimates for the forward process with no restraint based on the Jarzynski exponential average and on a sum of *two* Gaussian distributions. The two components of the mixture were determined with the ML-based expectation maximization algorithm (EM) [26, 27].

We used only two components as the EM fit notoriously becomes ill-defined with increasing number of components and with a low number of work values [28]. If the distribution is given by a mixture of $n_g$ normal distributions, $P(W)=\sum _i^{n_g} c_i n(\mu _i,\sigma ^2_i)$ , then the Crooks theorem allows to compute the free energy as: [25, 29]

6 $$\begin{aligned} \varDelta G_{EM}= -RT \ln \left[ \sum _i^{n_g} c_i e^{-\beta (\mu _i -\frac{1}{2}\beta \sigma _i^2)} \right] \end{aligned}$$

**Table 3:** Unidirectional estimates with no restraint (forward direction) for the binding free energies (kcal/mol) in the CB8-quinine system. No standard state correction applied. The 95% confidence intervals were calculated by bootstrapping with resampling from the 50 work values

$\tau$/ns	$\varDelta G_{\mathrm{Jarz.}}$	$\varDelta G_{EM_2}$
0.4	17.4 $\pm 0.5$	13.9 $\pm 3.9$
1.0	15.1 $\pm 0.6$	12.7 $\pm 3.9$
2.0	12.9 $\pm 1.1$	11.6 $\pm 2.7$

We can see in general that the estimate of the absolute dissociation free energy decreases with increasing time in all instances. This decrease is more pronounced for the Jarzynski exponential average. Due to the low number of work values, the apparently precise Jarzynski estimate at the longest NS time is likely to still exhibit a positive bias. We take hence the EM value at $\tau =2$ ns as the most reliable unbiased estimate for the A to B free energy change, albeit with still a rather large 95% confidence interval due to the limited number of work values.

## Conclusions

In Table 4 we finally compare the BAR-based bidirectional ABFE DSSB prediction with the Boresch-style restraint and the *forward* unidirectional EM estimate with no restraints. As previously noted, when enforcing the Boresch-style restraints during the A to B transition, the absence of the restraint potential in the A state prevents the cancellation of the entropy-related term, leading to an overestimation of the free energy of state B. This “degeneracy” term is not accounted for by Eq. 1. Noting that the Boresch-style restraints could have equivalently involved three host atoms on *any* of the eight symmetry-related glycoluril moieties with top or down configuration of the quinoline group, such missing entropic contribution to the ABFE can be roughly estimated as $T\varDelta S_{AB}= RT \ln (16) \simeq + {1.7}$ kcal/mol. This term is not present in the forward process with no restraints as the ligand is free to explore all equivalent poses at equilibrium in state A while the alchemical transitions proceed. The bias induced by the Boresch-style restraints could be even larger considering the reorganization energy due to the difference between of the restrained pose and the symmetrically related pose when no restraints are present (see Fig. 2). In the ESI we provide a forward estimate of this reorganization energy based on 50 NS transitions starting from state A where only the restraints gradually are enforced while the bound ligand is maintained in the fully coupled state.

**Table 4:** Bidirectional (restraints) and forward unidirectional (no restraints) ABFE estimates (units of kcal/mol)

	$N_w$	$t_{\mathrm{TOT}}$/ns	$\varDelta G$	$T\varDelta S$	$\varDelta G_{\mathrm{vol}}$	ABFE
BAR(bidirectional)	50	240	− 18.5 ± 0.8	1.7	7.5	− 9.3 ± 0.8
EM (unidirectional)	50	120	− 11.6 ± 2.7	n/a	4.5	− 7.1 ± 2.7

Going back to Table 2, we note that the unidirectional (A to B) EM estimate with no restraints is much less precise than the BAR estimate but appears to be closer to the experimental value of -6.5 kcal/mol. This fact could well be just a fortunate coincidence or may reflect the bias of the BAR estimate induced by the use of a set of imposed host-guest arbitrary restraints whose cost is (approximately) evaluated using the analytic correction of Eq. 1 plus the entropic contribution $T\varDelta S_{AB}$ and possibly a strain contribution due to a non optimal restrained pose (see Section 3 in the ESI). As the error in the EM estimates goes as $1/n^{1/2}$, we would need to run at least ten times more NS trajectories to bring the EM error to the level of that found when using BAR, unlikely approaching to the BAR estimate. In fact, from our experience [30], increasing the number of long-time trajectories has a direct impact on precision but a limited effect on the EM estimate. The unidirectional approach, by avoiding the enforcement of a user-defined restraint potential whose cost must be somehow evaluated, trades precision with accuracy.

In conclusion, we have seen that in the SAMPL6 SAMPLing NS-DSSB method the switching on (A to B) or off (B to a) the Boresch-style restraint potential is the main source of the observed huge dissipation of the NE processes for NS times as long as 2 ns. Such dissipation significantly affects the overlap (and hence the precision) in the BAR bidirectional estimate, and prevents the calculation of reliable *forward* unidirectional estimates even when collecting thousands of NS work values. The restraint potential has introduced a systematic and undetected bias (due to the entropy related term and to the strain energy) masked by the precision (reproducibility) of the BAR bidirectional estimate. *Stable* and unbiased unidirectional forward estimate are viable in the DSSB context if the restraint are weakened or even lifted altogether. In so doing, the standard state correction must be evaluated from the variance of the host-guest COM-COM distance in the equilibrium production run for state A, hence assessing the binding site volume $V_{\mathrm{site}}$. The unidirectional approach based on the forward process has pros and cons with respect to the bidirectional technique relying on the Boresch restraints used in the SAMPL6 SAMPLing submission. One benefit is that we need less simulation time and less work in general to obtain credible estimates (i.e. based on the character of the underlying work distribution) since we do not need to do the reverse process. This fact implies that we do not need to impose artificial strong restraints to keep the decoupling ligand in the *presumed* pose, with the danger of introducing an undetected bias. On the other hand, lifting the restraint *de facto* prevents the implementation of the precise BAR estimate, as the reverse process would produce highly dissipative trajectories with high probability, hence yielding a distribution with no overlap with the forward distribution even when resorting to high numbers of NS trajectories.

A possible workaround for the inherent lack of precision of unidirectional estimates has been recently proposed in Ref. [31], where the resolution of the forward work histograms was boosted by computing *separately* the decoupling and coupling work of the guest and combining the resulting histograms for the bound annihilation $P_b(W|A)$ and unbound growth $P_u(W|G)$ into the forward convolution $P(W|F)= (P_b*P_u)(W|F)= \int dw~P_b(W|A) P_u(W-w|G)$.

## Supplementary Information

Below is the link to the electronic supplementary material.

Electronic supplementary material 1 (PDF 1033 kb)

Electronic supplementary material 2 (ZIP 496 kb)
