# Supplementary material for: On the NS-DSSB unidirectional estimates in the SAMPL6 SAMPLing challenge
Source: J Comput Aided Mol Des. 2021 Oct 9;35(10):1055–65. doi: 10.1007/s10822-021-00419-0 (PMC8523005; doi:10.1007/s10822-021-00419-0)
Supplement: Supplementary file 1 — Electronic supplementary material 1 (PDF 1033 kb) [file 10822_2021_419_MOESM1_ESM.pdf]

Journal of Computer Aided Molecular Design manuscript No.
(will be inserted by the editor)

Supporting Information for "On the NS-DSSB unidirectional estimates in the SAMPL6 SAMPLing challenge"

Piero Procacci, Marina Macchiagodena

Received: date / Accepted: date

1 Effect of the Boresch-style restraints on the CB8 configurational average

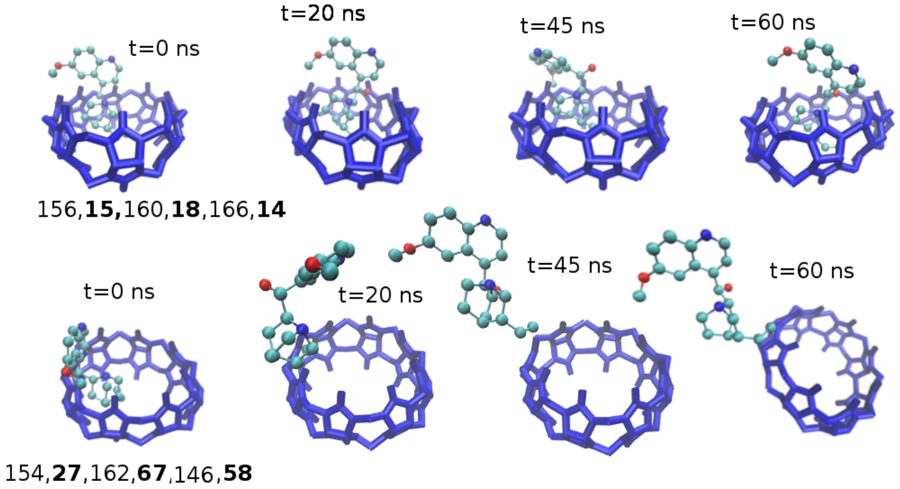

Fig. S1 Frames taken from a B state 60 ns MD simulation with the decoupled and restrained ligand in the CB8-quinine system. Top frames refer to Boresch-style restraints used in the SAMPL6 SAMPLing submission. Bottom frames refer to Boresch restraints that have been set by selecting 3 arbitrary CB8 and quinine atoms compatible with a state B configuration where the ligand is inside the CB8 torus. The `gro` file of such configuration is provided in the Supporting Information (SI). The indices of the six atoms for the restraints are reported below the t=0 ns structures. Top files for reproducing these data are provided in ESI compressed archive `ESI.zip`.

Piero Procacci
University of Florence, Department of Chemistry
Via Lastruccia n. 3, I-50019 Sesto Fiorentino (FI) (Italy)
Tel.: +39 055 4573081
E-mail: procacci@unifi.it

Boresch-style restraints should be imposed by selecting the three atoms on the host and the three atoms of the guest as part of relatively *rigid* moieties. It follows that, at variance with what is claimed in Ref. [1], the three ligand and three receptor atoms cannot be chosen "arbitrarily". Arbitrary choices of the three protein and ligand atoms (and hence of the six "relative" DOFs) according to the Boresch et al. recipes of Figure 2 on a given bound structure (or pose) could lead, during the dynamics, to relative ligand-receptor structures that are significantly different from the target pose. For example, as shown in Figure S1, if we choose *arbitrarily* the three host and guest atoms, setting the equilibrium distance and the angles of the six harmonic terms entering the Boresch function based on a single structure where the quinine guest is *inside* the CB8 torus host and then perform the MD simulation for the decoupled and host-restrained ligand, we may end sampling poses where the decoupled and restrained ligand is mostly *outside* the CB3 torus. So the "choice" of the six Boresch-style harmonic restraints (one distance, two bendings and three dihedral angles) to impose a specific ligand pose, far from arbitrary, is not, by any means, straightforward.

2 On the representativeness of the 50 selected starting points for the end-states A and B

In Figure S2a, we show the free energy surface (FES) with respect to the intramolecular CB8 coordinates corresponding to two orthogonal axes of the torus in the 5000 configurations sampled in the state A (i.e. when the unrestrained guest is fully coupled inside the CB8 torus). In Figure S2b we show the same FES for the configurations sampled in state B (restrained and decoupled ligand). The two CB8 internal coordinates are strongly anti-correlated as a stretching of one axis implies the shortening of the other orthogonal axis. Note also the effect of the ligand in modulating the FES. While the FES in the complex (state A) exhibits a perceptible banana-shape, the FES in state B shows no significant bending. A bending in the FES of state A can also be detected when using 50 frames sampled in a 20 ns simulation out of the total 5000 frames collected in the SAMPLing submission (for a total simulation time of 2 μs), indicating that these 50 starting points can be used as a meaningful representative set of the NS-DSSB SAMPLing for the following NS simulations.

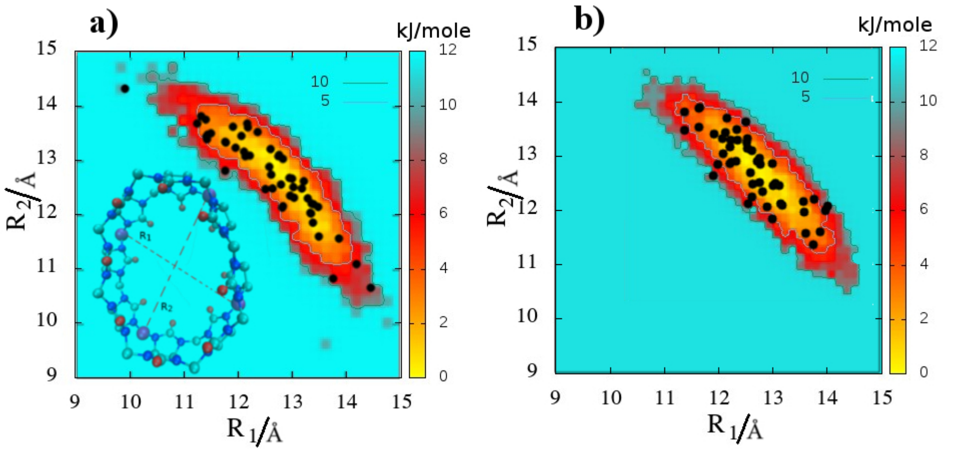

Fig. S2 FES with respect to two orthogonal distances in CB8 in a) the state A (CB8 with unrestrained and coupled quinine) and in) state B (CB8 with restrained and decoupled quinine). The black circles in state A and B refer to the 50 representative frames used in the NS runs.

In Figure S3, we show the probability distribution of the distance between the COM of the host and guest as obtained in the 5000 frames of states A and B and in the 50 corresponding representative frames. Remarkably, the (translational) binding site volume in the CB8-quinine system, as estimated[2] from the variance of the distribution as $V_{\rm site} = \frac{4}{3}\pi(2\sigma)^3$ in state A, is similar in the 50 and 5000 frames histograms, again an indication of the representativeness of these 50 frames.

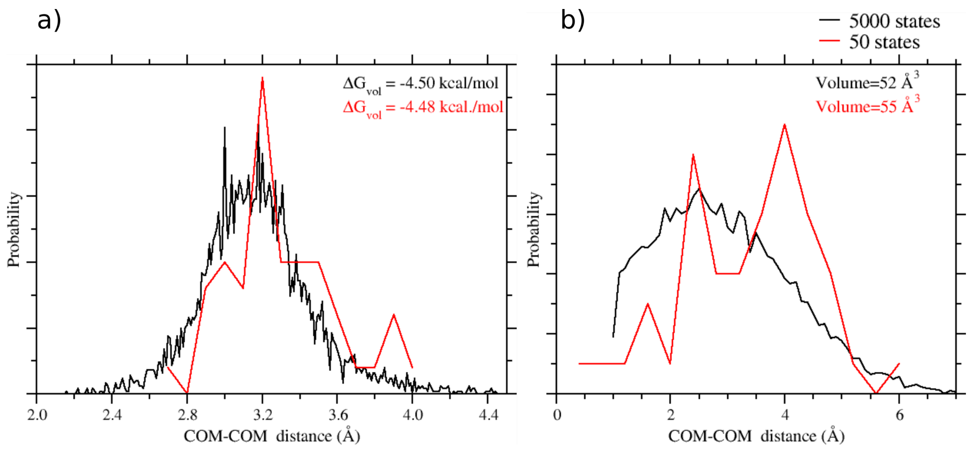

Fig. S3 CB8-quinine-guest COM-COM distance distributions in the state A with the fully coupled unrestrained guest (a) bound to the host and in the state B with decoupled guest bound to the host via the Boresch restraints (b). The standard state correction to the dissociation free energy in state A is computed as $\Delta G_{\rm vol} = -RT\ln(V_{\rm site}/V_0)$.

3 Bias in the Boresch-style restraint setup in the NS-DSSB SAMPL6 SAMPLing

In principle, if the restrained pose is selected so as to coincide with the actual ligand-receptor minimum in absence of restraints, the restraint work should be approximately independent on the restraint strength, in the assumption of harmonic oscillation of the ligand in the binding pocket. The work due to *switch on* a harmonic potential in state A while the ligand is maintained in the fully coupled state is in fact given by

$$\begin{aligned} W &= \int_0^\tau \frac{\partial H}{\partial t} dt = \int_0^\tau \frac{1}{2}\dot{K}(\zeta_i - \zeta_0)^2 dt \\ &= \frac{1}{\tau}\int_0^\tau \frac{1}{2}K(\zeta_i - \zeta_0)^2 dt \simeq \frac{1}{2}k_B T \end{aligned} \tag{1}$$

where $\dot{K} = \frac{K}{\tau}$ and ζ_i is an intermolecular restraint coordinate. The second equality in Eq. 1 follows assuming that the restrained pose coincides with that of the actual bound unrestrained complex. In this case the time average of the quadratic term is approximately equal $\frac{k_B T}{2}$. It follows that, in the ideal case, the cost of imposing the *six* harmonic Boresch restraints for a fully coupled ligand in the bound state should be equal to $3k_B T$ independently of the restraint strength.

In the NS-DSSB SAMPL6 SAMPLing setup, we have seen that in the forward (and reverse) direction, the total measured work of the driven process includes the entropic work due to the enforcement (release) of the restraints besides the "harmonic" term of $\simeq 3k_b T$. To asses the size of the bias in the NS-DSSB setup, we have computed the free energy change corresponding to the transition, starting from the state A, where only the restraints are enforced in $\tau = 1.0$ ns while the ligand remains fully coupled. The restraints were linearly switched on in a NS time $\tau = 2$ ns with K=1 and with K=10 producing 50 NS trajectories. The Jarzynski free energy estimates based the associated work distribution are reported in Table S1

K	$\Delta G_{\rm rstrs}$(Jar.)
1	4.5 ± 0.3
10	4.9 ± 0.2

Table S1 Free energy cost of imposing the Boresch-style restraints in the NS-DSSB setup in the state A with the fully coupled ligand. The Jarzynski estimates have been computed by collecting 50 NS transitions where the restraints where progressively imposed with the bound ligand always maintained in the fully coupled state

The bias work, $\Delta G_{\rm rstr}$, is much larger than $3RT$ and is (approximately) independent on the restraint strength and equal to $\simeq 4 : 5$ kcal/mol kcal/mol. This correction due to the Boresch restraints bias (entropy, strain and harmonic) would bring NS-DSSB binding free energy of the the CB8-quinine complex to -6:7 kcal/mol, in excellent agreement with the experimental value of -6.5 kcal/mol.[3]

References

1. S. Boresch, F. Tettinger, M. Leitgeb, and Ma. Karplus. Absolute binding free energies: A quantitative approach for their calculation. *J. Phys. Chem. B*, 107(35):9535–9551, 2003.
2. P. Procacci, M. Guarrasi, and G. Guarnieri. Sampl6 host–guest blind predictions using a non equilibrium alchemical approach. *J. Comput. Aided Mol. Des.*, Aug 2018.
3. S. Murkli, J. N. McNeill, and L. Isaacs. Cucurbit[8]urilguest complexes: blinded dataset for the sampl6 challenge. *Supramolecular Chemistry*, 31(3):150–158, 2019.
